# Brain network changes in adult victims of violence

**DOI:** 10.3389/fpsyt.2023.1040861

**Published:** 2023-02-02

**Authors:** Aliaksandra Shymanskaya, Nils Kohn, Ute Habel, Lisa Wagels

**Affiliations:** ^1^Department of Psychiatry, Psychotherapy, and Psychosomatics, Faculty of Medicine, RWTH Aachen University, Aachen, Germany; ^2^JARA-BRAIN Institute Brain Structure and Function, INM-10, Institute of Neuroscience and Medicine, Jülich Research Centre, Jülich, Germany; ^3^Donders Institute for Brain, Cognition, and Behavior, Radboud University Medical Center, Nijmengen, Netherlands

**Keywords:** victims of violence, neuroimaging, structural covariance, functional connectivity, partial correlation, sparse inverse covariance

## Abstract

**Introduction:**

Stressful experiences such as violence can affect mental health severely. The effects are associated with changes in structural and functional brain networks. The current study aimed to investigate brain network changes in four large-scale brain networks, the default mode network, the salience network, the fronto-parietal network, and the dorsal attention network in self-identified victims of violence and controls who did not identify themselves as victims.

**Materials and methods:**

The control group (*n* = 32) was matched to the victim group (*n* = 32) by age, gender, and primary psychiatric disorder. Sparse inverse covariance maps were derived from functional resting-state measurements and from T1 weighted structural data for both groups.

**Results:**

Our data underlined that mostly the salience network was affected in the sample of self-identified victims. In self-identified victims with a current psychiatric diagnosis, the dorsal attention network was mostly affected underlining the potential role of psychopathological alterations on attention-related processes.

**Conclusion:**

The results showed that individuals who identify themselves as victim demonstrated significant differences in all considered networks, both within- and between-network.

## 1. Introduction

The link between victimization and poor mental health has been recognized in many studies (1–3). Severe forms of victimization include physical and sexual violence. Additionally, different forms of abuse such as threat, stalking, blackmailing are often experiences as severe harm to an individual’s life (4, 5) including consequences such as depression, anxiety, suicidal ideation, and panic attacks. The self-identification as a victim thereby does not necessarily agree with external labeling (1) and the same event might be perceived very differently between individuals. Importantly, even if not ensured by external sources, self-perceived victimization is stressful and associated with negative consequences such as self-blame, loneliness, anxiety, and low self-worth (6). Further studies suggest that executive functioning is reduced in individuals who have experienced violence during early childhood or adolescence (7, 8). Given the severe and often protracted effects of perceived victimization, it is important to determine how this can lead to mental problems. In this respect, the brain plays an important role. However, while victimization represents a severe risk factor for mental disorders, only little is known with respect to victimization as a trans-diagnostic risk factor on the neural level.

Previous studies have investigated if the exposure to violence can affect brain morphology and brain function. Such associations between violence exposures and brain structural changes have been investigated for gray (GM) and white matter (WM). In GM, changes in volume (9–13), cortical thickness (CT) (12–16), surface area (12–14, 16), and local gyrification (14) were observed in connection to the experience of childhood neglect and abuse. Mostly GM and CT reductions were observed in victims of violence [e.g., (11–14)]. Nevertheless, increases were reported in female survivors of intimate partner violence (10). Additionally, GM volume reductions in the prefrontal cortex were reported (17), and these findings were recently confirmed in a trans-diagnostic sample of adult participants who reported childhood maltreatment. Another line of studies investigated neural changes in association to combat-related trauma (9, 18, 19). Interestingly these studies showed that combat exposure related volume reductions were distinct from reductions related to a PTSD and depression diagnosis (20, 21). In sum, structural abnormalities were observed in both cortical and subcortical regions in different samples in all tissues, although some findings (22) argue against a strong association of WM changes and the experience of violence. Furthermore, a recent study pointed to alterations in brain organization (23) based on the covariance of GM volume between selected areas in victims versus controls. Studies that explicitly focus on the subjective self-identification as a victim including a broad definition of violence in this field are lacking.

Changes in brain activity also have been associated with the exposure to violence (24–28). The majority of fMRI studies in different populations that had been exposed to violence showed deviations in activation during cognitive or emotional tasks [for a review see (29)], and functional connectivity alterations occurred during emotion provoking tasks (26, 30, 31) and resting state fMRI (32). Functional differences between survivors of intimate partner violence (IPV) with a PTSD diagnosis and a non-traumatized group were reported in the anterior insula, which is the hub of the salience network (26). Furthermore, decreased connectivity among the anterior insula, amygdala, and anterior cingulate cortex (ACC), was reported for IPV related PTSD during a face-match task (31). Moreover, painful stimulation led to an elevated activation of the right middle insula and the right dorsolateral prefrontal cortex in IPV survivors with PTSD (33). Potential PTSD specific influence may be expected here, since a decrease in subjective pain intensity ratings over time was accompanied by attenuation of activation within the right anterior insula, which at the same time was associated with avoidance symptoms of PTSD.

Previous results in survivors of violence were often specific to a certain age group or a specific type of violence. Many studies have focused on physical, sexual or emotional abuse experienced during childhood (34), underlining the role of the hippocampus and amygdala (17, 35–37). Furthermore, in populations that experienced violence in early childhood, physical forms of violence seem to be associated more strongly with changes in amygdala and anterior cingulate cortex while emotional abuse may result in changes related to reward and mood processing circuits (38). Other findings may even suggest differences in the brain networks of individuals exposed to emotion abuse versus neglect (39). While some studies have successfully shown changes in brain activation for specific victimized populations, brain changes have–to our knowledge–not been studied in transdiagnostic samples of individuals who identified themselves as victim including a broad definition of victimization.

The existing literature demonstrates the necessity to study the relationship between brain modulations and subjective victimization as a trans-diagnostic phenomenon, thereby enabling the identification of neural changes independent of a mental health diagnosis. Additionally, specific types of experienced violence have mostly been investigated in specific groups, for example combat related exposure in males and intimate partner violence in females. To our knowledge, currently there is no study that included male and female adults identifying themselves as victims independent of the type of violence, or age of the individual. Furthermore, only a few studies have so far investigated large-scale network changes simultaneously on a structural and functional level. Specifically, changes in the default mode network (DMN), the fronto-parietal network (FPN), and the salience network (SN) as well as the dorsal attention network (DAN) (40–42) have been proposed as prominent characteristics of psychiatric disorders and as markers of exposure to violence. Thus, studying changes in these networks and their association with previous victimization may support the identification of neural risk factors for mental health issues, independent of a specific diagnosis.

The current study aimed to identify differences in structural and functional covariance in the DMN, FPN, SN, and DAN in two different groups: The first group (V) was characterized by self-identification as victim of violence; the second group (NV) was matched to the V group by age, gender and the primary psychiatric diagnosis. Our study did not exclude participants based on the psychiatric diagnosis and represented therefore a more realistic clinical population, which enabled the investigation of structural and functional brain network connectivity. To investigate structural and functional organization differences, we focused on pre-determined regions of interest (ROIs) in the DMN, FPN, SN, and DAN, and analyzed group differences in between and within network covariance patterns of both function and structure.

We expected to discover differences in structural and functional organization of the four large-scale brain networks between V and NV, independently of any psychiatric diagnosis. Similar changes in covariance in SN and DMN were expected in the V group. We assumed, that presence of a psychiatric diagnosis played an additional important role in the difference between V and NV. Therefore, we performed a diagnosis-specific explorative analysis. As a secondary hypothesis, we assumed that the group of V with a present acute psychiatric diagnosis (V_D+_) differed from the NV with a present acute psychiatric diagnosis (NV_D+_), and a differing structural and functional covariance pattern would be observed as compared to the trans-diagnostic consideration. As a third hypothesis, we assumed, that V and NV would differ in their psychopathology, which in turn would correlate with the differences in the structural and functional covariance.

## 2. Materials and methods

### 2.1. Sample

The sample included two groups of adults of which the first group had subjectively experienced violence (V), while the matched control group had not experienced violence before (NV). Inclusion criteria for both groups were: (i) age between 18 and 60 years, (ii) right-handedness, (iii) MRI suitability, and (iv) absence of any neurological diseases. Specific inclusion criterion for the V group was the prior experience of at least one type of the following forms of violence. The experience of violence was verified by a screening instrument and a detailed qualitative interview which were developed within the “Gender Violence” project (43, 44). The definition of violence used applied to this screening instrument and the interview based on the WHO standards (45) defining physical, emotional, and sexual violence. Orienting to previous studies, and because it is a frequent precursor or other forms of violence during intimate partnerships (46), economic violence (financial abuse) was added as a further category in the screening. Physical forms included all forms of body attacks such as hitting, kicking, shaking, spitting; sexual forms include all sexual acts without agreement such as coercion, sexual assault or rape; emotional forms included permanent insults, humiliation, bullying, stalking, threat; economic forms included robbery, passing of salary, prohibition to fulfill basic needs. The NV group included only individuals who negated any prior experience of violence at a primary screening and did not identify themselves as victimized. The V group was recruited from the participant pool of a large study in which detailed semi-structured interviews about the experience of violence were performed. Within this larger study, participants in the V group were recruited on the one hand in cooperation with an intervention center against domestic violence in Aachen, Germany (“Frauen helfen Frauen e.V.”) who asked individuals with experiences of violence if they would be willing to participate in the study. Participation in the study was voluntary and completely independent of any further consultation. On the other hand, we distributed flyers describing different forms of violence, the study aims and contact points for individuals seeking help in all departments in the university hospital Aachen, including the emergency department and the psychiatric department. Individuals who self-identified themselves as victims of violence and wanted to participate could notify study personal *via* phone or email. Flyers were also distributed at other public places offering consultation or therapy to potential victimized individuals such as ambulant therapists. For individuals that were in addition to study participation or independent of study participation seeking help and that were not supported otherwise a team of trained experts and psychologists offered consultation as part of the project. The fMRI study only included individuals that had undergone the qualitative interview in the main arm of the study and a further screening concerning MRI criteria if participants were interested in taking part in the fMRI study. 33.3% of all recruited participants took part in the fMRI study as well. The NV group was directly recruited *via* flyers and at the university hospital RWTH Aachen, specifically the Department of Psychiatry, Psychotherapy and Psychosomatics. All participants gave their written informed consent to participate in the study and received a compensatory payment of 85 Euros. Included participants additionally underwent the Mini-International Neuropsychiatric Interview [MINI; (47)], which allowed us to match both groups for age, sex, and MINI diagnoses. Overall, the V group included 49 subjects and the NV group 41 individuals of which 25 in the V group and 20 in the NV group had any kind of psychiatric diagnosis.

### 2.2. Study protocol

The study protocol was approved by the internal Ethics Committee of the RWTH Aachen University and thus complied with the ethical principles stated in the Declaration of Helsinki. The complete study procedure consisted of an initial resting state fMRI scan, a social stress paradigm, an emotion induction paradigm, a second resting state scan, an anatomical scan, neuropsychological tests, and several self-report questionnaires.

Besides the behavioral variables, the present investigation focused on the anatomical scan and the first resting state scan. Imaging data were acquired on a whole-body Siemens 3T Trio scanner (Siemens AG; Erlangen, Germany) equipped with 12 channel head coil, located at the RWTH Aachen University hospital in Germany, whereas some subjects were measured after the scanner upgrade to Prisma. During the resting state acquisition, participants were instructed to relax and lie still with eyes opened, focusing a fixation cross presented on a black screen. Afterward, all participants assured that they had not fallen asleep.

In order to test if groups differed with regard to psychopathology severity, stress coping and neuropsychological functioning, after the MRI procedure, we quantified (i) the strength of depressive symptoms through the Beck Depression Inventory [BDI; (48)], (ii) state and trait anxiety scores through the State-Trait Anxiety Inventory [STAI; (49)], and (iii) information on stress exposure and stress symptoms through the Stress and Coping Inventory [SCI; (50)]. In the V group, we also measured perceived distress caused by violence experiences through the Impact of Event Scale [IES; (51)]. Neuropsychological tests included the digit span [ZNS, forward and backward; Hamburg Wechsler Intelligence test (HAWIE-R); (52)], the verbal fluency test [VLT; (53)], a measure for verbal intelligence [Mehrfach Wortschatztest version B, MWT_B; (54)] and a test for shared attention and executive functions/cognitive flexibility [Trail making test, TMT; (55)]. From the introduced neuropsychological tests, descriptive variables were derived: TMT comprised the difference between the acquired TMT version A and version B; VLT_1 represented the total fluency performance (i.e., phonemic fluency und semantic fluency), while VLT_2 represented switching (i.e., phonemic switching und semantic switching), and HAWIE-R (ZNS) represented the sum of the forward and backward digit-span tests.

Several participants could not be included in our analyses due to the following reasons: (i) missing anatomical (*n* = 4) or any resting state scans (*n* = 7) due to technical problems, (ii) incomplete coverage of the whole brain during structural scan (*n* = 10), (iii) influence of alcohol (*n* = 1), (iv) sudden nausea (*n* = 1), and (v) lack of credibility of statements due to several contradictions (*n* = 1). Thus, the final sample consisted of 64 participants, comprising 32 participants who experienced violence, and 32 controls. The number of V, who suffered from a current psychiatric diagnosis (V_D+_), was 25, and the number of V without a current diagnosis (V_D–_) was 7. The number of NV, who suffered from a current psychiatric diagnosis (NV_D+_), was 20, and the number of NV without a diagnosis (NV_D–_) was 12.

### 2.3. Voxel based morphometry

To investigate structural differences between both groups, we acquired a T1-weighed image for each participant using an MPRAGE sequence (TR = 2,300 ms, TE = 3.03 ms, flip angle = 9°, FOV = 256 × 256 mm^2^, 176 sagittal slices, voxel size = 1 × 1 × 1 mm^3^). Structural imaging data were preprocessed using the Computational Anatomy toolbox (CAT 12^1^). First, each scan was manually reoriented to the intercommisural plane. After correction for inhomogeneities in field intensity, affine and non-linear normalization to MNI standard space was applied using the DARTEL default template within a unified segmentation model (56). Then, images were segmented into GM, WM, and cerebrospinal fluid. Additionally, the GM volumes were scaled by the amount of contraction applied during the preceding normalization. This modulation with Jacobian determinants ensured that the total volume of GM corresponded to that of the original images. Finally, the modulated GM segments were smoothed using a Gaussian kernel of 8 mm FHWM which was suggested to improve the morphometric examination of smaller and larger brain regions (57, 58). A subsequent homogeneity check did not identify any outliers. The ensuing voxel-based morphometry data were used to examine covariance in GM volumes in the sample.

### 2.4. Functional resting state

To compare brain function between V and NV, 250 functional images for each participant were acquired using a EPI sequence (TR = 1,600 ms, TE = 30 ms, flip angle = 67°, FOV = 192 × 192 mm^2^, matrix size = 64 × 64, 26 transversal slices, voxel size = 3 × 3 × 4.2 mm^3^, acquisition order = interleaved ascending). Functional imaging data were preprocessed using the functional connectivity toolbox (CONN 18a^2^). Initially, the first four scans of each participant were discarded to allow for magnetic field saturation. Then, the individual resting state time series were preprocessed according to the following steps: (i) realignment and unwarping, (ii) slice-time correction, (iii) outlier detection [97th percentiles using Artifact Detection Toolbox (ART)], (iv) segmentation and spatial normalization to MNI standard space, and (v) smoothing (Gaussian kernel of 8 mm FWHM). Subsequently, the pre-processed time series were denoised to account for potential confounding effects of (i) 6 motion parameters, (ii) their derivatives, (iii) squares of the 6 motion parameters and their derivatives, (iv) mean CSF and WM signal (v) outlier regressors from ART. Additionally, quadratic detrending and despiking before regression were applied. We did not use global signal regression. Furthermore, the time-series were band-pass filtered to retain signals between 0.01 and 0.08 Hz. This frequency range likely represented neural signal and was less susceptible to physiological noise (59, 60). The resulting resting state time series were used to investigate functional connectivity in the sample.

### 2.5. ROI definition

**FIGURE 1:**
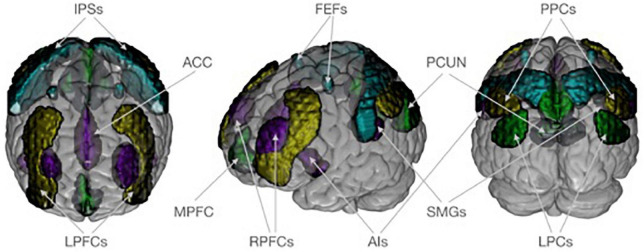
Neuroanatomical visualization of four target networks, according to CONN’s standard atlas. The default mode network is shown in dark green and consists of the medial prefrontal cortex (MPFC), the bilateral lateral parietal cortex (LPCs), and the precuneus (PCUN). The saliency network (purple) includes the anterior cingulate cortex (ACC) as well as the bilateral anterior insula (AIs), rostral prefrontal cortex (RPFCs), and supramarginal gyrus (SMGs). The dorsal attention network (turquoise) consists of frontal eye field (FEFs) and intraparietal sulcus (IPSs) bilaterally. The frontoparietal network (yellow) comprises both the right and left lateral prefrontal cortex (LPFCs) and posterior parietal cortex (PPCs). Regions of interest are plotted on an MNI standard brain in anterior, lateral, and posterior view using MRIcroGL (https://www.nitrc.org/projects/mricrogl/).

We were interested in how covariance within and between for major networks differed between V and NV. For that aim, the functional connectivity toolbox CONN was used (61). CONN’s standard network atlas was based on an independent component analysis of the functional resting state data of a large sample of healthy adults (61, 62). Although variances in the brain structure are expected in healthy controls and patient groups, applying the atlas information based on healthy adults for the investigation of patient groups is considered valid because previous studies have shown differences in the DNM, SN, and FPN based on different whole brain nodes and seeds [for a meta-analysis see Koch et al. (63)] suggesting robust group differences in these networks despite of potential structural differences. The atlas provides an established brain parcellation that divided the DMN, SN, DAN, and FPN into 19 spatially distinct network nodes, which were parts of the brain networks (Figure 1). The DMN covered the medial prefrontal cortex (MPFC), the bilateral lateral parietal cortex (LPCs), and the precuneus (PCUN). The SN included the anterior cingulate cortex (ACC) as well as the bilateral anterior insula (AIs), the rostral prefrontal cortex (RPFCs), and the supramarginal gyrus (SMGs). The DAN consisted bilaterally of frontal eye fields (FEFs) and the intraparietal sulci (IPSs). The FPN comprised both the right and left lateral prefrontal cortex (LPFCs) and the posterior parietal cortex (PPCs). The 19 investigated network nodes served as ROIs and were used to extract structural and functional brain information from individuals in the V and NV groups. For each participant, brain data was averaged across all voxels belonging to a particular ROI. This yielded individual average GM volumes, average GM density and average functional resting state time series for each ROI. The extracted GM volumes, densities and time series were z-standardized individually. This z-standardization mainly served two purposes in the following analyses: (i) to ensure the comparability of ROIs, and (ii) to enable the interpretation of covariance measures as correlation (= normalized covariance). To avoid potential confounding effects in the brain data, we accounted for sex, age, MINI diagnosis, antidepressants, and the number of other psychotropic drugs. In the structural analyses, we also accounted for total intracranial volume. Numerical confounds were z-standardized, while scale confounds were dummy encoded. Deconfounding on the group level was performed for time series in CONN, while for GM volumes and densities it was done with NiftiMapsMasker from nilearn package (64). The extracted network information served as input for the estimation of structural covariance and functional connectivity matrices in each group.

### 2.6. Sparse inverse covariance

To estimate covariance differences between the groups V/NV and V_D+_/NV_D+_, partial correlations between the 19 nodes were calculated on the group level. Group level partial correlations (assuming the group of subjects underlies the same functional or VBM structure) were calculated using sparse inverse covariance estimation [covariance precision from GraphicalLassoCV, from Python sklearn (64)]. By accounting for the influence of other brain regions, *partial* correlations as compared to *full* correlations yield direct, unbiased relationships between two ROIs (65, 66). Partial correlations could be estimated by sparse inverse covariance (67–69). An L1 penalty automatically set less important entries in the connectivity matrix to zero which enabled a robust estimation also in smaller samples (66, 69). The sparsity degree was internally chosen *via* a 3-fold cross-validation that ascertained the generalizability of the model to new data (70, 71). For calculations we used Python 3.7, primarily using the neuroimaging package nilearn (72) and the machine learning package scikit-learn (64).

To investigate the differences in structural covariance between V and NV, we estimated gray matter volumes (GMV), gray matter densities (GMD) and gray matter masses (GMM) of ROIs, and performed independent *t*-tests, to see if there are significant group differences between the values of GMD, GMV or GMM. Further, we investigated partial correlations in the estimated GM parameters between the 19 ROIs on group level separately in the V and NV groups. For this purpose, we used sparse inverse covariance estimation to determine the partial correlation between brain volumes on group level and reported the differences in covariance between V and NV. A single value in each subject for a given GM volume in each ROI prohibited estimation of individual partial correlation matrices.

**FIGURE 2:**
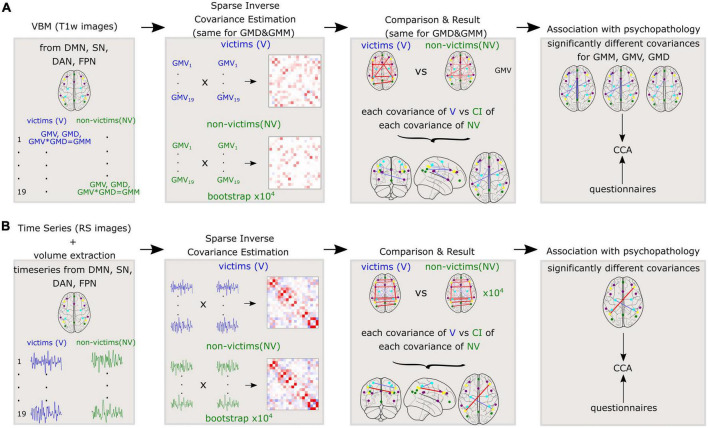
Methods applied to the whole cohort for **(A)**: Variables derived from gray matter (GM) such as gray matter volumes (GMV), gray matter densities (GMD), and gray matter masses (GMM) and **(B)**: Time series of resting state fMRI. Sparse inverse covariance was estimated between the 19 nodes on the group level. Further, the covariances were compared between V and NV. The differences in covariances were correlated with the questionnaires.

Afterward, we performed similar calculations for functional data, to investigate the differences in functional covariance between V and NV. An independent *t*-test probed for significant differences between the ROI time series of the V and NV groups. Again, sparse inverse covariance was used to generate partial correlations between brain volumes of each time series of resting state for each ROI. Analyses were performed separately for the V and NV groups. Described methods for GM variables and resting state are depicted in Figure 2.

The analysis steps described above for the determination of covariance differences, as well as statistical tests for group differences and brain-behavior associations, described in the following text, were repeated for a separate subgroup of subjects of the V and NV group with a current psychiatric diagnosis (D+). The subjects with a current diagnosis and an experience of violence (V_D+_) were compared to those with a current diagnosis but without an experience of violence (NV_D+_). These complementary calculations were performed to compare more homogeneous samples (see Tables 2, 3). Due to a small number of participant without a psychiatric diagnosis, we did not perform sub-analyses in these small V and NV groups.

**TABLE 1:** Mean and standard deviation of the subscales on the impact of events scale, the estimated duration of exposure to violence and the age at the first exposure to violence in the V group contrasting individuals who had experiences physical or sexual forms of violence to those who exclusively experienced other forms.

	Physical or sexual violence		No physical or sexual violence	
	*M*	SD	*M*	SD
Intrusion	18.33	9.096	13.33	9.552
Avoidance	20.95	9.870	19.78	8.700
Hyperarousal	16,15	10,520	13.78	9.615
Duration of exposure	3400.49	2996.61	2095.33	2124.99
Age at first exposure	11.83	9.62	24.33	17.54

**TABLE 2:** Sample characteristics for victims of violence (V) and non-victims (NV): Binary variables (sex, MINI diagnosis, and antidepressants): Statistical comparison of groups performed *via* chi-square test of independence; Continuous variables: mean ± standard deviation, statistical comparison of groups performed *via* independent two-sample *t*-test for normally distributed features [STAI trait, TMT, VLT_2_, HAWIE-R (ZNS)] and Mann Whitney *U*-test for non-parametric cases.

	V group (*N* = 32)	NV group (*N* = 32)	Group differences (*p*-values)
Sex	20♀ and 12♂	18♀ and 14♂	1.00
Age	33.3 ± 10.1	32.5 ± 11.7	1.00
BDI	15.5 ± 11.2	8.9 ± 10.4	0.033
STAI trait	46.1 ± 12.5	35.5 ± 16.3	0.060
SCI stress exposure	64.0 ± 19.8	47.9 ± 22.3	0.035
MINI diagnosis	78.1%	62.5%	1.00
Antidepressants	38.0%	25.0%	1.00
Number of other psychotropic drugs	0.2 ± 0.4	0.1 ± 0.4	0.588
Number of violence experiences	1.9 ± 0.9	—	
Childhood violence	1.3 ± 0.5	—	
TMT	−20.4 ± 12.5	−14.1 ± 10.6	0.232
VLT_1_	35.2 ± 8.6	39.4 ± 6.5	0.076
VLT_2_	29.5 ± 6.2	33.3 ± 4.8	0.101
HAWIE-R (ZNS)	14.9 ± 3.8	15.3 ± 3.8	1.00
MWT_B	28.3 ± 6.0	30.9 ± 3.2	0.113

Normality distribution was tested using Shapiro Wilk test. The equality of variance was tested with the Levene test. *P*-values were Bonferroni corrected at the significance level of 0.05.

**TABLE 3:** Sample characteristics for victims of violence (V_D+_) and non-victims (NV_D+_) with a current diagnosis: Binary variables (sex, MINI diagnosis, and antidepressants): Statistical comparison of groups performed *via* chi-square test of independence; Continuous variables: mean ± standard deviation, statistical comparison of groups performed *via* independent two-sample *t*-test for normally distributed features [SCI stress exposure, TMT, VLT_2_, HAWIE-R (ZNS)] and Mann Whitney *U*-test for non-parametric cases (the rest).

	V_D+_ group (*N* = 25)	NV_D+_ group (*N* = 20)	Group differences (*p*-values)
Sex	14♀ and 11♂	12♀ and 8♂	1.00
Age	33.2 ± 9.9	32.8 ± 12.0	1.00
BDI	17.3 ± 11.3	11.7 ± 10.9	1.00
STAI trait	47.0 ± 12.2	36.9 ± 18.9	0.263
SCI stress exposure	68.9 ± 18.8	53.3 ± 25.1	1.00
Antidepressants	36.0%	35.0%	1.00
Number of other psychotropic drugs	0.2 ± 0.4	0.2 ± 0.5	1.00
Number of violence experiences	2.0 ± 1.0	—	
Childhood violence	1.3 ± 0.5	—	
TMT	−21.6 ± 12.2	−12.5 ± 10.5	0.077
VLT_1_	33.6 ± 8.3	40.9 ± 7.2	0.022
VLT_2_	28.4 ± 6.2	33.5 ± 4.7	0.055
HAWIE-R (ZNS)	14.6 ± 3.9	14.7 ± 3.9	1.00
MWT_B	27.7 ± 6.5	30.7 ± 3.1	0.190

Normality distribution was tested using Shapiro Wilk test. The equality of variance was tested with the Levene test. *P*-values were Bonferroni corrected at the significance level of 0.05.

### 2.7. Group differences in covariance

Based on partial correlation maps, we examined group differences in covariance between ROIs. Negative values described a relative decrease in covariance, or cross-talk, between regions in the V group compared to the NV group. Complementarily, positive values described a relative increase of covariance, or higher level of cross-talk between the nodes. To inspect the observed structural and functional covariance patterns differences between victims and controls, we employed non-parametric test for mean differences (73, 74). To this end, we compared the data of V to the general distribution simulated by randomly drawing 10^4^ bootstrap samples of the NV, with replacement. Thus, every bootstrapped subsample consisted of 41 subjects for the NV group, and 20 in the NV group had any kind of psychiatric diagnosis. Structural and functional correlation matrices of the victims were compared to the bootstrapped 99.999% population intervals of the controls, which corresponds to testing for significant differences at a corrected, two-sided alpha-level of 10^–5^. The same analysis was performed for V_D+_ versus NV_D+_.

### 2.8. Summary of covariance differences across imaging modalities

To determine the convergence of network-specific covariance differences between the pairs of 171 nodes, we summed up the findings by calculating the frequency of differences in covariance between V and NV in every node across all imaging modalities. This number thus represented the total number of group differences in a network, to which these nodes belonged. The same was done separately for the V_D+_ versus NV_D+_ comparison. Since four networks were investigated, ten possible combinations for within- and between-network covariance existed. Four of those described the *within-network* covariance (DMN-DMN, SN-SN, DAN-DAN, and FPN-FPN), and the rest described the *between-network* covariance (DMN-SN, DMN-DAN, DMN-FPN, SN-DAN, SN-FPN, DAN-FPN). Additionally, we aimed to investigate the importance of differing covariance for a specific single node. To this end, the frequency of each node being involved in a different covariance comparing V versus NV and V_D+_ versus NV_D+_ was recorded.

### 2.9. Brain-behavior associations

Finally, significant structural and functional network aberrations were probed for their association with specific behavioral and neuropsychological variables. We used canonical correlation analysis (CCA) which investigated internal relationships between two sets of variables by seeking maximal correlations between combinations of variables in both sets (75, 76). Thus, the aim of the CCA was to test if a significant amount of variance of structural/functional network aberrations and behavioral/neuropsychological variables across subjects could be explained by pairs of canonical variates (modes of co-variation), and to discover the internal relationships between the two sets (74). The latter was done by the calculation of the correlations between each variable and the corresponding canonical variates.

For functional data, we estimated covariance between the nodes, which demonstrated significant differences between the subject groups, for each participant on the individual level. For functional data, we estimated covariance for each participant on the individual level. Statistical significance of canonical correlations was determined sequentially with a Wilks’ Lambda, using F-approximation (77). All *p*-values were Bonferroni-corrected to account for multiple comparisons and tested at a corrected alpha level of 0.05.

## 3. Results

### 3.1. Group characteristics

Within the V group experience of violence differed regarding the experiences type, length and age of exposure. In total, nine of 32 included participants in the V group experienced only emotional and economic violence and only one participant reported to have been exposed to economic violence as only form of violence. All other participants had experienced physical or sexual violence including multiple forms. Only 5 patients reported to have experienced physical violence without any other form of violence and only one participant reported to have been exposed to sexual violence only. Overall 22 participants reported to have been exposed to several forms of violence, while 10 reported only one form of violence. The estimated duration participants in the V group were exposed to one or more types of violence repeatedly was 8.3 years with only three individuals being exposed to violence (physical) only a single time. While the estimated mean duration did not differ significantly between individuals who had experienced physical or sexual violence (among others) and those who did not [*t*(30) = 1.19, *p* = 1.22, Table 1]. However, age of the first exposure was significantly lower individuals who experiences physical or sexual violence compared to those who did not [*t*(30) = 2.02, *p* = 0.035, Table 1]. Comparing the mean scores of the IES subscales intrusion, avoidance and hyperarousal of individuals who experienced (among others) physical or sexual violence in contrast (23) to those who did not report any of these forms (9) showed no significant differences in any scale [intrusion: *t*(28) = 1.36, *p* = 0.092; avoidance: *t*(28) = 0.31, *p* = 0.389; hyperarousal: *t*(28) = 0.58; *p* = 0.285]. Two participants (experiencing physical forms of violence) did not want to answer the IES, which is why the mean scores are reported only for a group of 30 participants.

As shown by non-significant group differences, we were able to successfully match self-identified victims and the control group in age, sex, and MINI diagnoses (Table 2). The groups did not significantly differ in any neuropsychological variable or questionnaire.

Comparison of groups only including participants with a current psychiatric diagnosis (V_D+_ and NV_D+_) were provided in Table 3. The groups did not significantly differ in any neuropsychological variable or questionnaire. Furthermore, no significant differences were discovered neither between GMM, GMV or GMD nor between the time series in ROIs of the V and NV groups and the V_D+_ and NV_D+_ groups.

### 3.2. Structural covariance in brain networks in all subjects, independent on the psychiatric diagnosis

**FIGURE 3:**
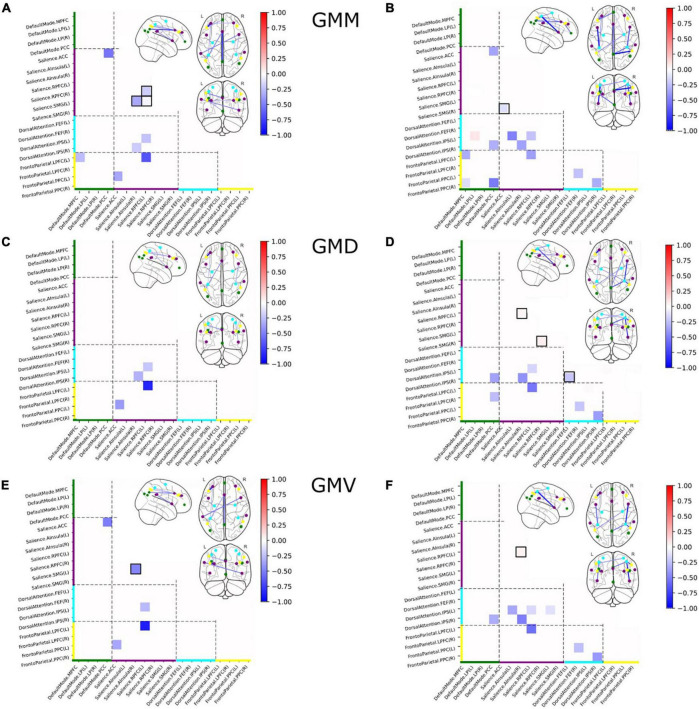
Group differences in structural covariance of gray matter masses (GMM) **(A)**, gray matter densities (GMD) **(C)**, and gray matter volumes (GMV) **(E)** within and between four major brain networks for the full sample, and group differences in structural covariance of GMM **(B)**, GMV **(D)**, and GMD **(F)** within and between four major brain networks for the subsample of subjects with a current psychiatric diagnosis. Squares indicate significant differences in partial correlations between V and NV. Colors on the axes and of the nodes correlated with the networks: DMN–green, SN–purple, DAN–cyan, FPN–yellow. Nodes with within-network differences were highlighted with black squares.

Based on the structural covariance matrix, and after performing previously described bootstrapping to identify differences in covariance between V and NV, we identified 9 out of 171 node pairs, that demonstrated differences in GMM covariance between groups at a corrected alpha level of 10^–4^ (Figure 3A). Significantly less covariance between regions in V was observed in all significantly different covariance measures. *Within-network* disturbances emerged only in the SN and constituted 33% of all detected aberrations, demonstrating less covariance and therefore lower homogeneity in the structural organization of V as compared to NV. *Between-network-wise*, FPN, DAN, SN, and DMN revealed aberrations in half of their nodes. On the other hand, GMD (Figure 3C) demonstrated 4 aberrant connections, all of which overlapped with GMM covariance differences. These consistent aberrations were observed in SN/DAN and SN/FPN. GMV (Figure 3E) demonstrated 5 aberrant connections, which showed reduced covariance in V, same as in GMM. These aberrations were observed in GMM, and occurred within SN, in SN/FPN, SN/DMN, and SN/DAN.

### 3.3. Functional covariance in brain networks in all subjects, independent of psychiatric diagnosis

**FIGURE 4:**
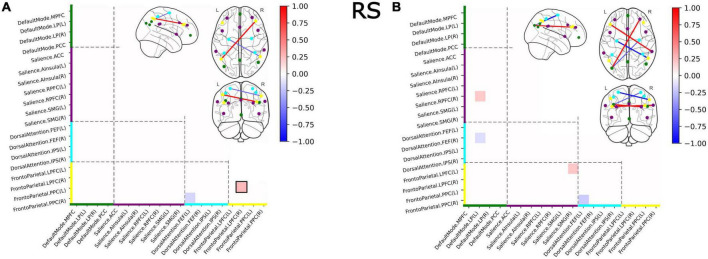
Group differences in functional covariance of RS within and between four major brain networks for the full sample **(A)** and for the subsample of subjects with a current psychiatric diagnosis **(B)**. Squares indicate significant differences in partial correlations between victims and controls. Colors on the axes and of the nodes correlated with the networks: DMN–green, SN–purple, DAN–cyan, FPN–yellow. Nodes with within-network differences were highlighted with black squares.

The comparison of functional covariance matrices yielded 2 out of 171 functional connections that significantly differed between groups at a corrected alpha-level of 10^–4^ (Figure 4A). Significantly *higher* covariance between regions in V was observed *within* the FPN network. *Between-network-wise*, less covariance was observed in V in FPN/DAN covariance.

We furthermore investigated differences in structural and functional covariance between groups with a current MINI diagnosis (V_D+_ and NV_D+_).

### 3.4. Structural covariance in brain networks in subjects with a current psychiatric diagnosis

We identified 13 out of 171 pairs of nodes (Figure 3B) that differed significantly in GMM between V_D+_ and NV_D+_. Only one node (DMN/DAN) showed a slight overexpression in V_D+_, as compared to NV_D+_. Lower covariance between regions in V_D+_ was observed in the remaining 12 connections. Within-network disturbances emerged only in the SN. Between-network-wise, the aberrations were observed in DMN, FPN and DAN across all modalities. GMV (Figure 3D) demonstrated 10 aberrant connections, which showed less covariance in V_D+_. Within-network covariance aberrations were observed in SN, and in DAN. In contrast to the whole sample GMV analysis (V/NV), group differences in the V_D+_/NV_D+_ sample demonstrated a decrease in DAN covariance with DMN and FPN. Furthermore, GMD demonstrated 9 aberrant connections, with significant decrease in covariance in DAN/DMN and DAN/FPN, and with within-network covariance aberrations in SN (Figure 3F).

### 3.5. Functional covariance in brain networks in subjects with a current psychiatric diagnosis

The comparison of functional covariance matrices yielded 4 out of 171 functional connections, that significantly differed between the V_D+_ and NV_D+_ groups at a corrected alpha-level of 10^–4^ (Figure 4B). Covariance in V_D+_ differed from NV_D+_ in the same four nodes as in the V versus NV group, and in the three additional nodes. In these nodes, SN/DMN and SN/FPN demonstrated higher, and DAN/DMN lower covariance in V_D+_ as compared to NV_D+_. No *within-network* differences were observed.

### 3.6. Across-modality covariance differences

**FIGURE 5:**
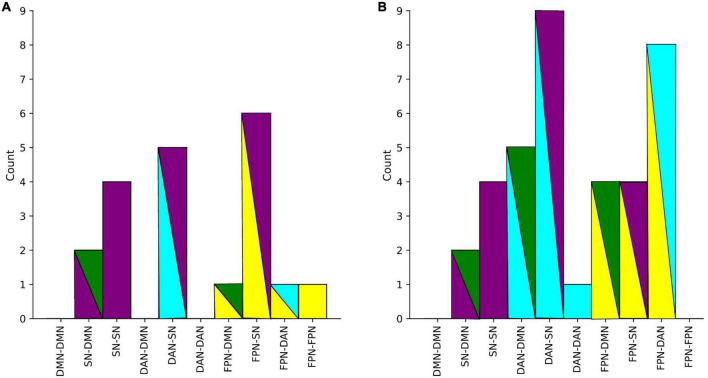
**(A)** Number of all significant group differences in covariance between networks in the main sample. **(B)** Differences in covariance between networks in D+ sample (subjects with a psychiatric diagnosis). Colors represent networks: DMN–green, SN–purple, DAN–cyan, FPN–yellow.

**FIGURE 6:**
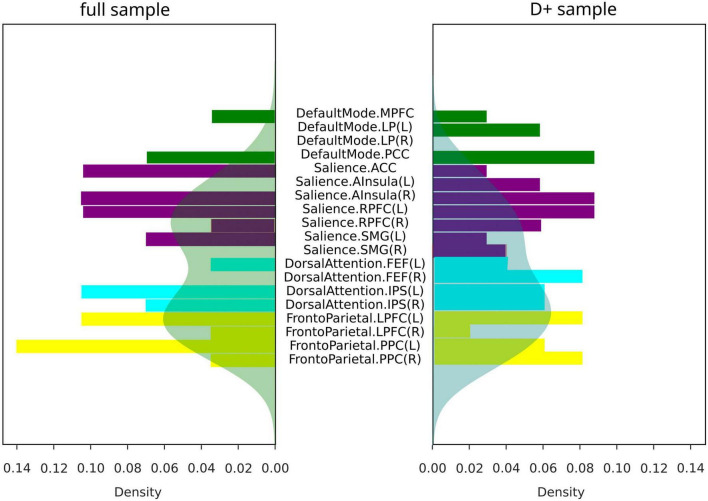
Covariance differences across modalities in all subjects (full sample for V/NV comparison, and D+ sample for V_D+_/NV_D+_ comparison). Colors represent networks: DMN–green, SN–purple, DAN–cyan, FPN–yellow. Shaded areas represented density functions of the histograms and highlighted differences in frequency of node involvement.

The summaries of structural and functional covariance differences between the two V and NV groups across all modalities were depicted in Figure 5A for V versus NV, and in Figure 5B for V_D+_ versus NV_D+_. The histograms demonstrated that covariance in V_D+_ was to a higher degree different from NV_D+_, than in the analogous comparison of the V versus NV. Specifically, this meant that the networks in V_D+_ were less covariant between each other, than in NV_D+_. In particular, regarding the V_D+_ versus NV_D+_ comparison, the DAN was associated with the majority of within- and between-network covariance differences (33% of all differences), followed by the SN (28%). In contrast, the main sample (V versus NV) showed mostly differences in the SN (49% of all differences), followed by the FPN (26%). Upon characterizing the affected network nodes on the individual level (Figure 6), we demonstrated that the DMN and DAN nodes were affected to a lower degree in the main sample than in the D + sample (10 versus 14% for DMN and 20 versus 27% for DAN). On the other hand, SN and FPN were affected more in the full sample (40 versus 32% for SN and 30 versus 27% for FPN).

### 3.7. Association with psychopathology

**FIGURE 7:**
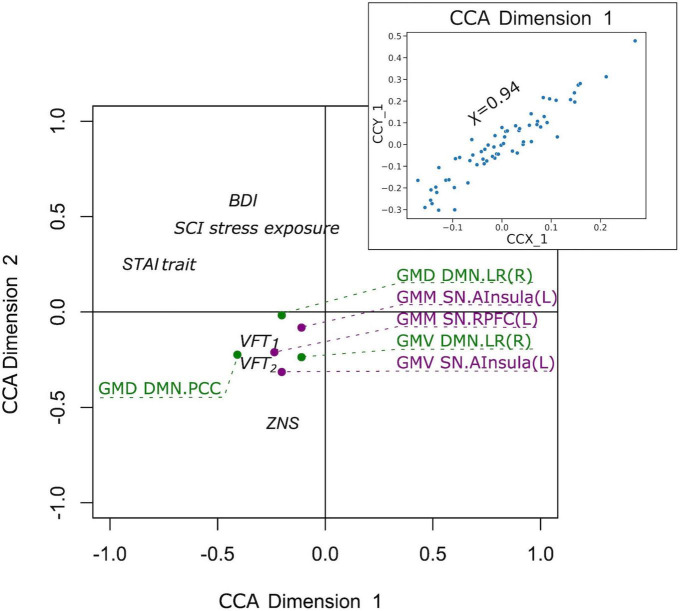
Correlation of the first canonical correlation analysis (CCA) mode variables for the full sample (upper right), and contributions of the variables to the first and second CCA dimensions.

Finally, we examined if the observed group differences in structural and functional covariance were related to psychopathological symptoms (STAI trait, BDI, SCI stress exposure), as well as to neuropsychological functions [VFT_1_ with one, and VFT_2_ with two categories, TMT, MWT and HAWIE-R (ZNS)] using CCA. In the full sample (V versus NV), the analysis revealed a single highly significant CCA mode that related brain connectomes to subject measures (*r* = 0.94, *p* = 0.008). We observed that 94% of the variation in brain connectomes was explained by the variation in questionnaires. Since only the first CCA mode was significant, the first canonical variate for brain measures (CCX_1) was plotted against the first canonical variate for the questionnaires (CCY_1) in the scatter plot (Figure 7). These correlations between each variable and the corresponding canonical variate were used to interpret the first CCA mode, and the correlations with the correlation over *r* > 0.2 were provided in Table 4. The contributions of the variables to the CCA modes were also demonstrated in Figure 7. All correlations between the first canonical variable for brain, and the brain measures were uniformly large, and were represented by all included measures of DMN and SN. Among the psychopathological symptoms and neuropsychology variables, STAI contributed to CCY_1 to the highest proportion. Thus, CCY_1 can be considered as an anxiety measure. Thus, due to the significance of the CCA decomposition, CCX_1 and CCY_1 demonstrated high correlation, and uncovered dependence between anxiety traits and a linear combination of structural brain measures of SN and DMN. However, the CCX_1 and CCY_1 did not differ significantly between V and NV. Thus, the latent variables did not reflect the victimization status.

**TABLE 4:** Correlation between canonical correlation analysis (CCA) variates and variables in the full sample.

Brain measure variable	Correlation with CCX_1
GMD DMN.LP (R)	−0.21
GMD DMN.PCC	−0.30
GMM SN.AInsula (L)	−0.20
GMM SN.RPFC (L)	−0.23
GMV DMN.LP (R)	−0.20
GMV SN.AInsula (L)	−0.21
**Questionnaire variables**	**Correlation with CCY_1**
BDI	−0.27
STAI trait	−0.83
SCI stress exposure	−0.39
HAWIE-R (ZNS)	−0.28
VFT_1_	−0.25
VFT_2_	−0.29

**FIGURE 8:**
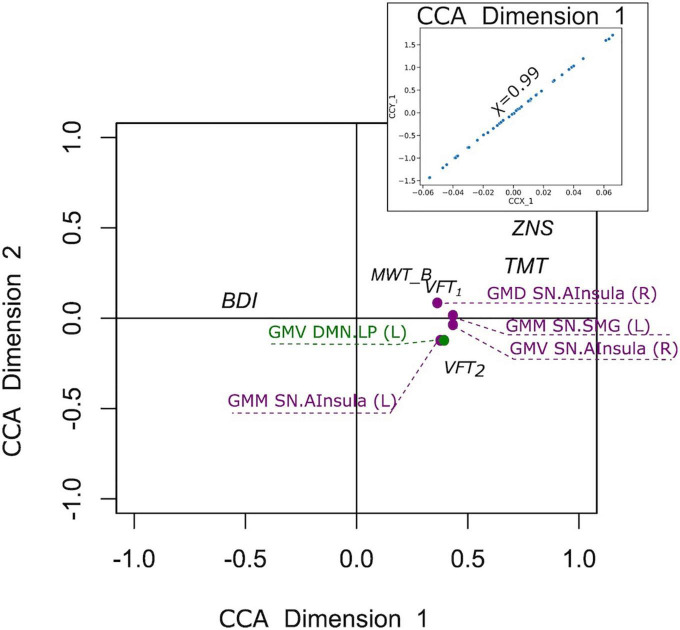
Correlation of the first canonical correlation analysis (CCA) mode variables for the sample of subjects with a current psychiatric diagnosis (upper right), and contributions of the variables to the first and second CCA dimensions.

In the D+ groups, the number of subjects was not sufficient to estimate the modes of variance reliably for both RS and GM brain measures. However, based on the previous analysis of the full sample, we considered for the CCA analysis only GM brain measures. The analysis revealed a single highly significant CCA mode that related brain connectomes to subject measures (*r* = 0.99, *p* < 10^–5^). Again, the first canonical variate for brain measures (CCX_1) was plotted against the first canonical variate for the questionnaires (CCY_1) in the scatter plot (Figure 8). The highest correlations between each variable and the corresponding canonical variate were provided in Table 5. The contributions of the variables to the CCA modes were also demonstrated in Figure 8. The correlations between the first canonical variable for brain, and the brain measures were uniformly large, and were again represented by the measures of DMN and SN. Therefore, the canonical variate CCX_1 could again be considered as an overall measure across all brain measures. On the other hand, TMT and ZNS contributed to CCY_1 to the highest proportion. Thus, the dependence between TMT and ZNS, and a linear combination of structural brain measures of SN and DMN, was discovered.

**TABLE 5:** Correlation between canonical correlation analysis (CCA) variates and variables for the sample of subjects with a current psychiatric diagnosis.

Brain measure variable	Correlation with CCX_1
GMV DMN.LP (R)	0.42
GMV SN.AInsula (R)	0.43
GMD SN.AInsula (R)	0.39
GMM SN.AInsula (L)	0.41
GMM SN.SMG (L)	0.43
**Questionnaire variables**	**Correlation with CCY_1**
BDI	−0.36
MWT_B	0.26
TMT	0.68
HAWIE-R (ZNS)	0.71
VFT_1_	0.44
VFT_2_	0.57

## 4. Discussion

The current study aimed to identify differences in structural and functional covariance in the DNM, FPN, SN, and DAN in a trans-diagnostic sample of individuals who identified themselves as victims of violence. This sample was compared to individuals who did not indicate any prior experience of violence but had a similar history of mental disorders. To further limit the influence of different psychopathologies on the differences in network covariance in both groups, two comparisons were made: a comparison of victims and non-victims in the whole sample (V versus NV), and in a subsample with the present psychiatric diagnosis (D+ group: V_D+_ versus NV_D+_). Applying multiple comparisons correction, the only differences between V and NV was discovered in BDI and SCI stress exposure, both higher in V. On the other hand, the only difference between V_D+_ versus NV_D+_ was discovered in VLT_1, which was higher in NV. These differences may be linked to the exposure to violence indirectly as the patients in this study who mostly had experienced violence over a long time and with multiple incidents may have had an increased severity of emotional and cognitive symptoms compared to other patients who may not have had any traumatic experiences.

While no differences between the V and NV group nor between V_D+_ and NV_D+_ group were observed neither in GMM/GMV/GMD nor in the time series in ROIs, the relative organization of the brain seemed to be different between groups. Specifically, differences in structural and functional covariance within and between the four selected networks were discovered. Sparse inverse covariance of the GM parameters and RS time series between the regions showed both positive and negative partial covariance differences within and between networks in both the full sample comparisons, and in the D+ sample comparisons.

Differences in the covariance in all four investigated networks, detected in V versus NV, may reflect organizational differences in the brain of victimized individuals related to specific characteristics of the group. However, we did not find any correlation between observed neural differences and the self-reports. There may be different explanations: on the one hand, self-reports may have not reliably reflected well-being and psychopathological symptoms due to the influence of self-perceptual abilities and social desirability. On the other hand, differences in network organization may have had heterogeneous sources or were related to further variables not specifically assessed in this study. While single values of psychopathological symptoms and neuropsychology did not differ in comparisons of V versus NV and V_D+_ versus NV_D+_, CCA analysis discovered significant CCA modes in both cases. This way, the questionnaires of the full sample, mainly represented by anxiety, related to the brain measures of DMN and SN. On the other hand, neural measures of the DMN and SN in the D+ sample explained the variability in shared attention and executive functions/cognitive flexibility as well as working memory, based on the uncovered single significant CCA mode. Evidence was found for the relationship between working memory and executive functioning, which might point to the common executive attention construct (78). While no direct evidence was found, the strongly affected covariance of the DAN might underlie the observed CCA modality in the D+ sample. Nevertheless, the latent variables did not reflect the victimization status, since the CCX_1 and CCY_1 did not differ between V_D+_ and NV_D+_.

Structural and functional differences did not show a large overlap, which may further support the heterogeneous sources of variance in the self-identified victims and non-victims. In patients with major depression that experienced childhood trauma disturbances in functional brain networks similar to those investigated in our study have been associated with trauma severity (79). Higher childhood trauma severity moreover predicted symptoms of anxiety which may show some similarity to the association of the anxiety related component and covariance measures of gray matter in SN and DMN. In addition to factors that directly relate to the exposure of and severity to violence, cultural influences, personality, genetics, and for the patients in both groups also access and success of mental health treatment, may contribute to the reorganization of brain structure and function. These different sources of variance may impact structure and function differently thereby concealing or enhancing organizational changes in structure or function. Our results underline what has been summarized in a systematic review on subtypes of violence and associated functional and structural alterations; deviations occur in different brain regions not only depending on the subtype of violence but also with regard to structure and function activity and connectivity or integrity (39). The biological pathway the authors suggest may be one reason for such differences. Early-life stress (exposure to violence) is expected to affect brain organization which then may result in functional network changes either directly or rather indirectly accompanying pathology. Instead of originally affected stress-related brain regions, regions that are associated with other cognitive processes affected by dysfunctional stress systems may show functional disturbances in later life. Observing such a mismatch of structural and functional covariance measures may thus support the independence or asymmetry of structural and functional network organization. The SN was affected in both the full sample and the subjects with a current diagnosis (D+), with it being the most affected network in the full sample. Structurally, in the full sample and in the D+ sample, the SN demonstrated *reduced* within- and between-network covariance. Functionally, however, *no differences* relative to NV were observed in the full sample, while in V_D+_ SN demonstrated *increased* covariance in SN/DMN and SN/FPN. Thus, V_D+_ in our study demonstrated functional hyperconnectivity of SN, which was not observed in victims if the group also included healthy, potentially more resilient individuals. It could be therefore hypothesized, that especially those victims with a present diagnosis exhibited a functionally disturbed SN. Nevertheless, the victimized group proved to suffer from structural aberrations in the SN covariance. In healthy populations, the SN has been recognized as necessary for the efficient regulation of activity in the DMN. Thus, the failure of this regulation would lead to inefficient cognitive control and weaker performance on cognitive control tasks (80). Correspondingly, although in our sample patients in the V and NV group were mostly not free of a psychiatric diagnosis, possibly especially the victim group suffered from loss of control. Similarly, in Bogliacino et al. (81), a reduction of cognitive control in victims of urban violence and warfare was reported. Furthermore, differences involving SN in the D+ sample were most frequent in DAN/SN, while in the full sample this was the case for the FPN/SN covariance. The latter network communication seems to be responsible for the externally directed cognition (80). Finding altered communication between FPN/SN in the whole V sample may thus underline that these regions are affected independent of the severity or violence or the mental health consequences.

Node connections of the DMN also demonstrated major differences between V and NV in both structure and function. The DMN in healthy subjects is responsible for a self-referential introspective state (82). Structurally, both the full sample and the D+ sample demonstrated *reduced* between-network covariance in DMN/FPN. DMN/FPN connection was shown to be responsible for introspective processes and executive function (83). Thus, the reduced structural covariance between DMN and FPN might point to possible *decreased* introspective processes, often observed in victims elsewhere (83). While this would have to be confirmed in future studies, such a deficit might be more pronounced in participants in the V_D+_ group due to an active psychiatric diagnosis. Functionally, the inverse interplay between psychopathology and large-scale brain networks of the DMN/FPN has been demonstrated before (41) for a number of psychiatric diagnoses. This might explain the observed higher number of differences in DMN/FPN structural covariance as compared to the full sample in our study. Functionally, however, we could not support this finding. While no differences were discovered in the full sample, V_D+_ demonstrated reduced covariance in DMN/DAN (i.e., the connection DMN.PCC/DAN.IPS), and increased covariance in DMN/SN. DMN/DAN covariance has been related to perceptual attention in healthy populations (83), while in anxiety and PTSD patients, functional covariance impairments were observed in dorsolateral prefrontal cortex (84). While DMN/DAN covariance seemed to be similar between V and NV, we cannot make a definitive statement in this regard in the D+ sample due to opposite covariance between different nodes. More research into the single nodes is required at this point.

The DAN is engaged during externally directed attentional tasks (85). In V and V_D+_, the DAN demonstrated *reduced* within-and between-network covariance both in structure and function. Additionally, to the DAN/SN and DAN/DMN differences in covariance, described above, D+ sample demonstrated a higher number of differences in structural covariance in DAN/FPN. FPN regulates DAN in accordance with goals and task demands, and it is involved in the regulation of perceptual attention (83). Recent data showed negatively associated network connectivity between DAN and FPN in subjects with depression, anxiety and suicidality (41). Thus, based on our findings and in line with others, the higher proportion of DAN/FPN covariance differences in D+ sample might be a sign of the less efficient attention processes as compared to the full sample.

In interpreting these findings, several limitations have to be taken into account. First, only selected networks were investigated, therefore differences in other networks cannot be excluded. These networks were anatomically defined which may introduce a larger bias than extracting data-driven time series as in other studies (86). The victims were self-identified victims of violence, which is a highly subjective measure, and it cannot be quantified, since no correlation between aberrant network nodes and behavioral variables were discovered. Nevertheless, it is important to investigate neural alterations related to the subjective perception as this perception may be strongly connected to mental health problems (6). Despite of attempts to account for a large heterogeneity with regard to psychopathology by matching both groups with regard to the primary psychiatric diagnosis, the total sample was too small to test different subgroups with specific disorders and subgroups without any mental disorder. In addition, the heterogeneity of the type of violence individuals were exposed to was large and the size of the sample did not allow us to test in network changes may differ depending on specific forms of violence such as (exclusively social) or non-social forms of violence. As pointed out in a recent review (38) physical and sexual violence in early childhood may seems to be associated with higher risks of PTSD and personality disorders while emotional violence more often associated with developing major depression. Animal models of physical versus non-physical abuse even suggest that brain circuit changes associated with abuse may differ. The current results, referring to the in changes of brain connectivity across all different types of violence may therefore conceal more specific changes associated with physical or non-physical violence. Further research in single nodes and in subgroups must be performed, while the study sample is to be extended. Finally, the upgrade of the scanner to Prisma while the study was carried may have introduced data variance which can reduce the classification accuracy in the data as shown in projects applying classifiers on fMRI data in multi-side projects (87).

In a nutshell, differences in functional and structural covariance between self-identified victims and people who never experienced violence or did not identify themselves as victims were observed, with a primary role of the SN. In the group with heightened pathologies and various mental disorders, most differences between victims and non-victims occurred in DAN. When the sample was controlled for psychiatric disorders, less covariance differences were observed, indicating that a major part of the network variance may reflect differences in the pathological status of two groups.

## Data availability statement

The raw data supporting the conclusions of this article will be made available by the authors, without undue reservation.

## Ethics statement

The studies involving human participants were reviewed and approved by Internal Ethics Committee of the RWTH Aachen University. The patients/participants provided their written informed consent to participate in this study.

## Author contributions

LW, NK, and UH designed the study. LW collected the data. AS and LW preprocessed and analyzed the data and wrote the manuscript. All authors provided feedback and approved the final manuscript version.

## Footnotes

1
www.neuro.uni-jena.de/cat

2
www.nitrc.org/projects/conn
